# CASP8 -652 6N Del Polymorphism Contributes to Colorectal Cancer Susceptibility: Evidence from a Meta-Analysis

**DOI:** 10.1371/journal.pone.0087925

**Published:** 2014-02-03

**Authors:** Qiliu Peng, Xianjun Lao, Weizhong Tang, Zhiping Chen, Ruolin Li, Jian Wang, Yan Deng, Taijie Li, Xue Qin, Shan Li

**Affiliations:** 1 Department of Clinical Laboratory, First Affiliated Hospital of Guangxi Medical University, Nanning, Guangxi, China; 2 Department of Anal and Colorectal Surgery, First Affiliated Hospital of Guangxi Medical University, Nanning, Guangxi, China; 3 Department of Occupational Health and Environmental Health, School of Public Health at Guangxi Medical University, Nanning, Guangxi, China; 4 Department of Medicine Research, First Affiliated Hospital of Guangxi Medical University, Nanning, Guangxi, China; Northwestern University Feinberg School of Medicine, United States of America

## Abstract

**Objective:**

Caspase-8 (CASP8) plays a central role in the apoptotic pathway and aberrant regulation of this pathway may cause cancers. Previous studies investigating the association between CASP8 -652 6N ins/del polymorphism and colorectal cancer (CRC) risk showed inconclusive results. We performed a meta-analysis of all available studies to investigate this association.

**Methods:**

All studies published up to October 2013 on the association between CASP8 -652 6N ins/del polymorphism and CRC risk were identified by searching electronic databases PubMed, EMBASE, and Cochrane library. The association between CASP8 -652 6N ins/del polymorphism and CRC risk was assessed by odds ratios (ORs) together with their 95% confidence intervals (CIs).

**Results:**

Six studies with 6,325 cases and 6,842 controls were included in the meta-analysis. We observed that the CASP8 -652 6N ins/del polymorphism was significantly correlated with CRC risk when all studies were pooled into the meta-analysis (ins/del vs. ins/ins: OR = 0.890, 95%CI 0.821–0.964, *P* = 0.004; del/del + ins/del vs. ins/ins: OR = 0.899, 95%CI 0.833–0.970, *P* = 0.006). In stratified analyses by ethnicity, source of control, and quality score, significant association was observed in Asians (ins/del vs. ins/ins: OR = 0.862, 95%CI 0.761–0.977, *P* = 0.020; del/del + ins/del vs. ins/ins: OR = 0.845, 95%CI 0.749–0.953, *P* = 0.006), population-based studies (ins/del vs. ins/ins: OR = 0.890, 95%CI 0.813–0.975, *P* = 0.012; del/del + ins/del vs. ins/ins: OR = 0.901, 95%CI 0.827–0.982, *P* = 0.018), and high quality studies. However, in subgroup analysis according to cancer location, no significant association was detected.

**Conclusions:**

The present meta-analysis suggests that the CASP8 is a candidate gene for CRC susceptibility. The CASP8 -652 6N ins/del polymorphism may play a protective role in CRC development especially among Asians. Further large and well-designed studies are needed to confirm this association.

## Introduction

Colorectal cancer (CRC) is the second most commonly diagnosed cancer with over 1.2 million new cases and 608,700 deaths in 2008 [1], [2]. The highest incidence rate of CRC is found in Australia, Europe, and North America. In addition, the incidence rate of CRC is rapidly increasing in a number of countries within Eastern Asia, such as China [2]. The development and progression of CRC involves unregulated epithelial cell proliferation due to a series of accumulated genetic alteration [3]. Evidence has shown that prolonged survival of such genetically unstable colorectal epithelial cells, leading eventually to their ultimate malignant transformation, is associated with progressive inhibition of apoptosis. Genetic polymorphisms for genes controlling cell cycle or apoptosis have been found to modulate the risk for human malignancies [4], [5].

Caspase-8 (CASP8) is a key regulator of apoptosis. It is an apical protease of the extrinsic apoptosis pathway that plays an important role in defense mechanism against hyper-proliferation and tumorigenesis [6]. The human CASP8 gene, mapped to chromosome 2q33–34, is 30 kb in length and contains at least 11 exons [7]. There were at least 474 single nucleotide polymorphisms in the CASP8 gene according to the dbSNP database (http://www.ncbi.nlm.nih.gov/SNP), including the most commonly occurring CASP8 −652 6N ins/del polymorphism (rs3834129). It was reported that the CASP8 −652 6N ins/del promoter variant destroy the binding element for stimulatory protein 1 and reduce the expression of CASP8, thus resulting in a reduction in the apoptosis reactivity of T lymphocytes upon stimulation by cancer cells [8]. Hence, it is biologically reasonable to hypothesize a potential relationship between the CASP8 −652 6N ins/del polymorphism and cancers.

Over the last two decades, several molecular epidemiological studies have evaluated the association between CASP8 −652 6N ins/del polymorphism and CRC risk, but the results remain controversial and inconclusive. For genetic association case-control studies that check candidate polymorphisms, sample size is an important influencing factor for study accuracy. Small sample size might have insufficient power to explore a true associations of modest effect [9], especially for complex multifactorial disease such as CRC. Combining data from all eligible studies by meta-analysis has the advantage of increasing statistical power and reducing random error and obtaining precise estimates for some potential genetic associations. Therefore, in this study, we conducted a quantitative meta-analysis including all eligible studies. This is, to our knowledge, the first comprehensive meta-analysis of genetics studies on the association between CASP8 −652 6N ins/del polymorphism and CRC risk.

## Materials and Methods

### Search strategy

A literature search of Pubmed, Embase and Cochrane library databases was conducted using the combined keywords: ‘CASP8’, ‘Caspase8’, ‘polymorphism’, ‘genetics’, ‘colon cancer’, ‘rectal cancer’ and ‘colorectal cancer’. The latest search was done in October 2013, without any language restriction. Additional articles were identified through the references cited in the first series of articles selected. Articles included in the meta-analysis were in any language, with human subjects, published in the primary literature and had no obvious overlap of subjects with other studies. Among overlapping reports, only the studies with more information on origin of cases/controls were retained. The study was performed according to the proposal of Meta-analysis of Observational Studies in Epidemiology group (MOOSE) [10].

### Selection criteria

The following criteria were used to include published studies: (i) Case–control studies which evaluated the association between CASP8 −652 6N ins/del polymorphism and CRC risk; (ii) sufficient genotype data were presented to calculate the odds ratios (ORs) and 95% confidence intervals (95% CIs); (iii) control population did not contain malignant tumor patients. Major reasons for exclusion of studies were (i) review, or meta-analysis, or letter, or comment; (ii) duplicated studies, or studies without raw data we need; and (iii) studies that focused on HNPCC or FAP. Family–based studies of pedigrees with several affected cases per family were also excluded, because their analysis is based on linkage considerations.

### Data extraction

Two authors (Qiliu Peng and Xianjun Lao) independently reviewed and extracted data from all eligible studies. To ensure the accuracy of the extracted information, the two authors checked the data extraction results and reached consensus on all of the data extracted. If different results were generated, they would check the data again and have a discussion to come to an agreement. If these two authors could not reach a consensus, another author (Weizhong Tang) was consulted to resolve the dispute and a final decision was made by the majority of the votes. Data extracted from eligible studies included the first author, year of publication, country of origin, ethnicity, genotyping method, matching criteria, source of control, CRC diagnosis criteria, total numbers of cases and controls and genotype frequencies of cases and controls. Ethnic backgrounds were categorized as Caucasian, and Asian. Cancer location was divided into colon cancer and rectum cancer and was additionally recorded for the stratified analysis.

### Quality score assessment

The quality of eligible studies was evaluated independently by two authors (Qiliu Peng and Xianjun Lao) according to a set of predefined criteria (Table 1) based on the scale of Thakkinstian et al. [11]. The revised criteria cover the representativeness of cases, source of controls, ascertainment of CRC, total sample size, quality control of genotyping methods, and Hardy-Weinberg equilibrium (HWE) in the control population. Disagreements were resolved by consensus. Scores ranged from 0 (lowest) to 10 (highest). Articles with scores equal to or less than 6 were considered “low-quality” studies, whereas those with scores higher than 6 were considered “high-quality” studies.

**Table 1:** Scale for Quality Assessment.

Criteria	Score
Representativeness of cases	
Selected from cancer registry or multiple cancer center sites	2
Selected from oncology department or cancer institute	1
Selected without clearly defined sampling frame or with extensive inclusion/exclusion criteria	0
Source of controls	
Population or community based	2
Both population-based and hospital-based/healthy volunteers/blood donors	1.5
Hospital-based controls without colorectal cancer	1
Cancer-free controls without total description	0.5
Not described	0
Ascertainment of colorectal cancer	
Histological or pathological confirmation	2
Diagnosis of colorectal cancer by patient medical record	1
Not described	0
Sample size	
>1000	2
200–1000	1
<200	0
Quality control of genotyping methods	
Clearly described a different genotyping assay to confirm the data	1
Not described	0
Hardy-Weinberg equilibrium	
Hardy-Weinberg equilibrium in controls	1
Hardy-Weinberg disequilibrium in controls	0.5
No checking for Hardy-Weinberg disequilibrium	0

### Statistical analysis

The strength of the association between CASP8 −652 6N ins/del polymorphism and CRC risk was assessed by odds ratios (ORs) with 95% confidence intervals (CIs). The significance of the pooled OR was determined by Z test and a *p* value of less than 0.05 was considered significant. The association of CASP8 −652 6N ins/del polymorphism and CRC risk was assessed using additive models (del/del vs. ins/ins and ins/del vs. ins/ins), recessive model (del/del vs. ins/del + ins/ins), and dominant model (del/del + ins/del vs. ins/ins). Heterogeneity among studies was checked by a chi-square-based Q-test [12]. A *P_Q_* value less than 0.10 for the Q-test indicates a presence of heterogeneity among studies, and so the random-effects model (the DerSimonian and Laird method) was used for the meta-analysis [13]. Otherwise, the fixed-effects model (the Mantel–Haenszel method) was used [14]. To explore the sources of heterogeneity among studies, we performed subgroup analyses and Galbraith plots analysis. Subgroup analyses were performed by ethnicity, cancer location, source of control, and quality score. Sensitivity analysis was performed by sequential omission of individual studies to assess the robustness of the results. Publication bias was evaluated using a funnel plot and Egger's regression asymmetry test [15]. If publication bias existed, the Duval and Tweedie non-parametric “trim and fill” method was used to adjust for it [16]. The distribution of the genotypes in the control population was tested for HWE using a goodness-of-fit Chi-square test. All analyses were performed using Stata software, version 12.0 (Stata Corp., College Station, TX). All *p* values were two-sided. To ensure the reliability and the accuracy of the results, two authors entered the data into the statistical software programs independently with the same results.

## Results

### Study characteristics

Based on the search criteria, eight studies relevant to the role of CASP8 −652 6N ins/del polymorphism on CRC susceptibility were identified. Two of these articles were excluded: one was a letter [17], one did not present sufficient data for calculating OR and 95% CI [18]. Manual search of references cited in the published studies did not reveal any additional articles. As a result, a total of six relevant studies containing 6,325 cases and 6,842 controls were included in the meta-analysis [8], [19], [20], [21], [22], [23] (Figure S1). Table 2 lists the main characteristics of these studies. Among these publications, two were conducted in Caucasian descent [20], [21], and four were conducted in Asian descent [8], [19], [22], [23]. Three were population–based studies [8], [21], [22] and three were hospital–based studies [19], [20], [23]. Two of these studies [19], [22] presented CASP8 −652 6N ins/del polymorphism genotype distributions according to cancer location (colon cancer and rectal cancer). The cases were histologically or pathologically confirmed as CRC in four studies [19], [20], [22], [23]. Controls were mainly healthy or hospital-based populations and matched with age and gender. The genotype distributions in the controls of all studies were in agreement with HWE.

**Table 2:** Characteristics of studies included in the meta-analysis.

First author (Year)	Country	Ethnicity	Sample size (case/control)	Genotyping methods	Matching criteria	Source of control	CRC confirmation	HWE(*P* value)	Quality scores
Wu 2013	China	Asian	451/631	PCR-SSCP	Age	HB	Patho-	0.119	8
Theodoropoulos 2011	Greece	Caucasian	402/480	PCR-RFLP	Age and gender	HB	Histo-	0.194	6
Pittman 2008	UK	Caucasian	3,879/3,661	CAS-PCR	Age, gender and region	PB	NA	0.170	8
Sun 2007	China	Asian	918/890	PCR-RFLP	Age and gender	PB	NA	0.116	7
Liu 2010	China	Asian	370/838	PCR-RFLP	Age, gender, smoking and drinking	PB	Histo-	0.538	8.5
Xiao 2013	China	Asian	305/342	PCR-PAGE	Age and gender	HB	Histopatho-	0.905	5

CRC, Colorectal cancer; Histopatho-, Histopathologically confirmed; Histo-, Histologically confirmed; Patho-, Pathologically confirmed; NA, Not available; PB, Population–based; HB, Hospital–based; HWE, Hardy–Weinberg equilibrium in control population; PCR–RFLP, Polymerase chain reaction-restriction fragment length polymorphism; PCR-SSCP, Polymerase chain reaction-single strand conformation polymorphism; CAS-PCR, Competitive allele-specific PCR; PCR-PAGE, Polymerase chain reaction-polyacrylamide gel electrophoresis

### Meta-analysis

**Figure 1:**
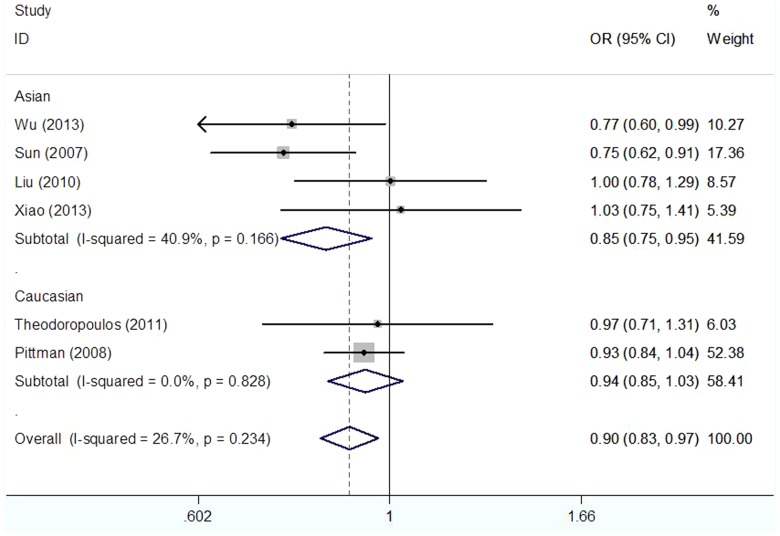
Forest plot of the CASP8 −652 6N del polymorphism and CRC risk using a fixed-effect model (additive model ins/del vs. ins/ins).

**Figure 2:**
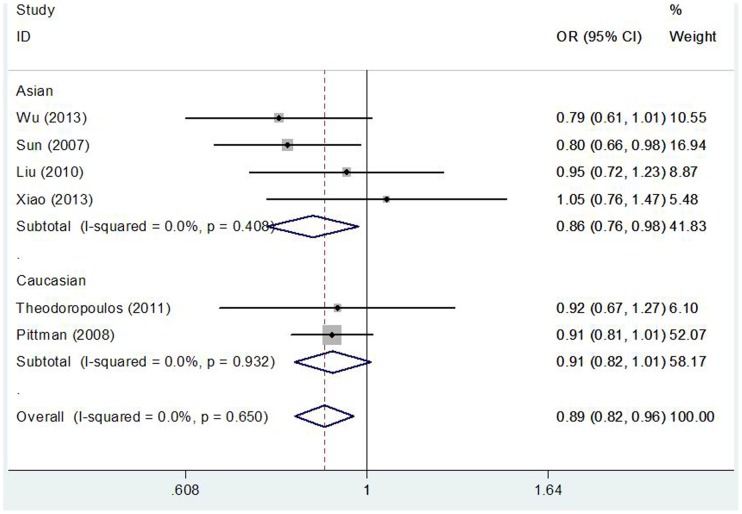
Forest plot of the CASP8 −652 6N del polymorphism and CRC risk using a fixed-effect model (dominant model del/del + ins/del vs. ins/ins).

As shown in Table 3, We found that the CASP8 −652 6N ins/del polymorphism was significantly correlated with decreased CRC risk when all studies were pooled into the meta-analysis (ins/del vs. ins/ins: OR = 0.890, 95%CI 0.821–0.964, *P* = 0.004; del/del + ins/del vs. ins/ins: OR = 0.899, 95%CI 0.833–0.970, *P* = 0.006). In subgroup analysis by ethnicity, significant decreased CRC risk was found in Asian populations (ins/del vs. ins/ins: OR = 0.862, 95%CI 0.761–0.977, *P* = 0.020, Figure 1; del/del + ins/del vs. ins/ins: OR = 0.845, 95%CI 0.749–0.953, *P* = 0.006, Figure 2), but not in Caucasian populations. In stratified analysis according to source of control, significant decreased CRC risk was found in population-based studies (ins/del vs. ins/ins: OR = 0.890, 95%CI 0.813–0.975, *P* = 0.012; del/del + ins/del vs. ins/ins: OR = 0.901, 95%CI 0.827–0.982, *P* = 0.018), but not in hospital-based studies. In subgroup analysis by quality score, significant decreased CRC risk was observed in high quality studies (ins/del vs. ins/ins: OR = 0.877, 95%CI 0.805–0.956, *P* = 0.003; del/del + ins/del vs. ins/ins: OR = 0.886, 95%CI 0.817–0.961, *P* = 0.004), but not in low quality studies. However, in subgroup analysis by cancer location, statistical significant association was not detected in both colon cancer patients and rectum cancer subjects.

**Table 3:** Meta-analysis of CASP8 −652 6N ins/del polymorphism and risk of CRC.

Analysis	No. of studies	del/del vs. ins/ins (Homozygote)		ins/del vs. ins/ins (Heterozygote)		del/del + ins/del vs. ins/ins (Dominant model)		del/del vs. ins/del + ins/ins (Recessive model)	
		OR (95% CI)	*P/P_Q_*	OR (95% CI)	*P/P_Q_*	OR (95% CI)	*P/P_Q_*	OR (95% CI)	*P/P_Q_*
Overall	6	0.884(0.672–1.164)	0.380/0.026	**0.890(0.821–0.964)**	**0.004**/0.650	**0.899(0.833–0.970)**	**0.006**/0.234	0.943(0.733–1.212)	0.644/0.028
Ethnicity									
Caucasian	2	0.995(0.882–1.123)	0.939/0.668	0.910(0.819–1.010)	0.077/0.932	0.937(0.849–1.034)	0.198/0.828	1.060(0.960–1.170)	0.250/0.642
Asian	4	0.782(0.465–1.317)	0.356/0.028	**0.862(0.761–0.977)**	**0.020**/0.408	**0.845(0.749–0.953)**	**0.006**/0.166	0.817(0.500–1.336)	0.421/0.041
Cancer location									
Colon	2	0.869(0.460–1.643)	0.667/0.301	0.821(0.629–1.071)	0.146/0.552	0.827(0.641–1.069)	0.146/0.405	0.932(0.496–1.753)	0.828/0.345
Rectum	2	1.065(0.645–1.760)	0.806/0.239	0.877(0.702–1.096)	0.248/0.294	0.898(0.726–0.112)	0.326/0.118	1.115(0.678–1.832)	0.668/0.156
Source of control									
HB	3	0.925(0.684–1.250)	0.611/0.402	0.890(0.750–1.055)	0.179/0.369	0.890(0.755–1.048)	0.163/0.304	1.008(0.774–1.312)	0.955/0.373
PB	3	0.887(0.536–1.468)	0.641/0.004	**0.890(0.813–0.975)**	**0.012**/0.515	**0.901(0.827–0.982)**	**0.018**/0.109	0.938(0.579–1.519)	0.794/0.005
Quality score									
>6	4	0.836(0.552–1.266)	0.387/0.007	**0.877(0.805–0.956)**	**0.003**/0.543	**0.886(0.817–0.961)**	**0.004**/0.122	0.891(0.599–1.325)	0.568/0.008
≤6	2	1.027(0.728–1.447)	0.880/0.568	0.985(0.783–1.239)	0.896/0.566	0.997(0.800–1.242)	0.976/0.784	1.087(0.812–1.454)	0.575/0.445

CRC, Colorectal cancer; CASP8, Caspase 8; *P_Q_*, P values of Q-test for heterogeneity test; OR, odds ratio; CI, confidence intervals; HB, Hospital–based studies; PB, Population-based studies

### Heterogeneity analysis

**Figure 3:**
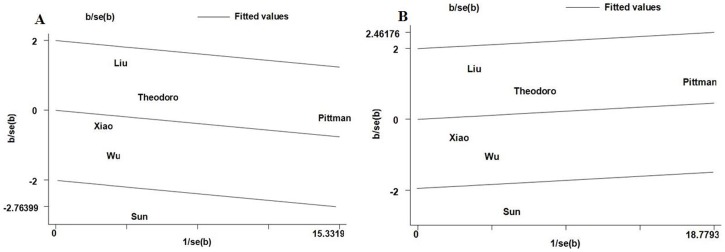
Galbraith plots analysis of CASP8 −652 6N del polymorphism and CRC risk. A The study of Sun et al. was spotted as outlier in additive model ins/del vs. ins/ins. B The study of Sun et al. was spotted as outlier in recessive model del/del vs. ins/del + ins/ins.

Statistical significant heterogeneity among studies was observed when all eligible studies were pooled into the meta-analysis (del/del vs. ins/ins: *P_Q_* = 0.026; del/del vs. ins/del + ins/ins: *P_Q_* = 0.028). To explore the sources of heterogeneity, we first performed subgroup analyses. Subgroup analyses stratified by ethnicity, source of control, and quality score showed that the heterogeneity was still significant in Asian populations, population-based studies, and high quality studies (Table 3). Subsequently, we performed Galbraith plots analysis to further identify the source of heterogeneity. Galbraith plots analysis indicated that the study Sun et al. [8] was the outlier contributing to the heterogeneity in additive model del/del vs. ins/ins and recessive model del/del vs. ins/del + ins/ins in the overall populations (Figure 3). When excluding the study by Sun et al. [8], the heterogeneity decreased obviously and *P_Q_* values were greater than 0.10 in the overall populations (del/del vs. ins/ins: *P_Q_* = 0.414; del/del vs. ins/del + ins/ins: *P_Q_* = 0.454), Asians (del/del vs. ins/ins: *P_Q_* = 0.153; del/del vs. ins/del + ins/ins: *P_Q_* = 0.182), population-based studies (del/del vs. ins/ins: *P_Q_* = 0.170; del/del vs. ins/del + ins/ins: *P_Q_* = 0.212), and high quality studies (del/del vs. ins/ins: *P_Q_* = 0.168; del/del vs. ins/del + ins/ins: *P_Q_* = 0.219). However, the significance of the summary ORs for CASP8 −652 6N ins/del polymorphism in different comparison models in the overall population and subgroup analyses were not influenced by omitting this study [8].

### Sensitivity analysis

Sensitivity analysis was performed by sequential omission of individual studies. For analyses of pooling more than three individual studies, the significance of ORs was not influenced excessively by omitting any single study (data not shown), indicating that our results were statistically robust.

### Publication bias

**Figure 4:**
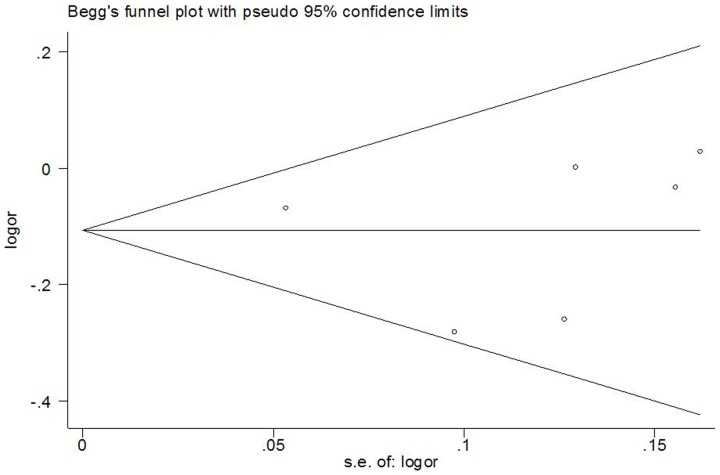
Funnel plots for publication bias of CASP8 −652 6N del polymorphism and CRC risk in the overall populations (dominant model del/del + ins/del vs. ins/ins).

Begg's funnel plot and Egger's test were performed to assess the publication bias of literatures in all comparison models. The shape of the funnel plot did not reveal any evidence of obvious asymmetry (Figure 4). Then, the Egger's test was used to provide statistical evidence of funnel plot symmetry. All the p values of Egger's tests were more than 0.05 (P = 0.561 for del/del vs. ins/ins; P = 0.929 for ins/del vs. ins/ins; P = 0.476 for recessive model del/del vs. ins/del + ins/ins; and P = 0.912 for dominant model del/del + ins/del vs. ins/ins), providing statistical evidence of the funnel plots' symmetry. The results suggested that publication bias was not evident in this meta-analysis.

## Discussion

Programmed cell death, or apoptosis, is a normally protective process that enables the body to eliminate harmful cells. Impaired apoptotic mechanisms can lead to uncontrolled cell proliferation and result in the pathogenesis of human cancer [24]. Caspase-8, which was encoded by the CASP8 gene, plays a central role in the apoptotic pathways [25] and changes in the genetically determined structure of this enzyme can influence the rate of apoptosis. More specifically, a six-nucleotide deletion polymorphism (−652 6N del) has been identified in the promoter region of the CASP8 gene and is associated with decreased RNA expression in lymphocytes due to the altering of an Sp1 binding site [8]. This variant has been found to decrease CASP8 activity and apoptotic reactivity of T lymphocytes through the cancer cell ex vivo model [8], and the decreased CASP8 activity may lead to an alteration of normal programmed cell death and result in tumor susceptibility. This hypothesis was confirmed by our meta-analysis.

Our meta-analysis results showed that the CASP8 −652 6N ins/del polymorphism was associated with a reduced risk of CRC among the Asian population. However, no significant association was detected among the Caucasian population. In addition, our data also showed a decreased CRC risk under the additive model (ins/del vs. ins/ins) and dominant model (del/del + ins/del vs. ins/ins) in the overall populations. When we excluded the study of Sun et al. [8], which was shown as an outlier in Galbraith plots analysis, a statistically significant decreased CRC risk was also found in Asian population but not in Caucasians under the additive model and dominant model. Actually, it might not be uncommon for the same polymorphism play different roles in cancer susceptibility among different ethnic populations. In Caucasians, the differences in genetic backgrounds and the environment they lived in may influence the association between the CASP8 −652 6N ins/del polymorphism and CRC risk. In addition, the limited number of studies also makes the results from subgroup analysis by ethnicity less reliable. Thus, our results should be interpreted with caution.

In subgroup analysis according to the source of control, statistically significant decreased CRC risk was found in the population-based studies but not in hospital-based studies. The reason may be that the hospital-based studies have a high risk of producing unreliable results because hospital-based controls may not always be truly representative of the general population. When stratified according to the quality score of the articles, statistically significant decreased CRC risk was observed in high quality studies but not in low quality studies. The possible reason for the discrepancy may be that the existence of selection bias and recall bias in the studies of lower quality. In addition, genotyping methods without quality control in studies of low quality should be also considered when deciphering these inconsistent results. Therefore, a methodologically preferable design, such as using a proper and representative population-based high quality study, is of great value in case–control studies.

Heterogeneity is a potential problem when interpreting the results of a meta-analysis, and finding the sources of heterogeneity is one of the most important goals of meta-analysis [26]. In the present study, significant between-study heterogeneity in the pooled analyses of total eligible studies was observed in additive model del/del vs. ins/ins (*P_Q_* = 0.026) and recessive model del/del vs. ins/del + ins/ins (*P_Q_* = 0.028). To explore the sources of heterogeneity, we performed subgroup analyses and Galbraith plots analysis. Subgroup analyses stratified by ethnicity, source of control, and quality score showed that the heterogeneity was still significant in Asian populations, population-based studies, and high quality studies. Galbraith plots analysis showed that the study Sun et al. [8] was the outlier in the two genetic models in the overall populations. When excluding the study Sun et al. [8], the heterogeneity decreased obviously and all *P_Q_* values were greater than 0.10 in the two genetic comparison models in the overall populations, Asians, population-based studies, and high quality studies. However, the summary ORs in additive model del/del vs. ins/ins (*P_Q_* = 0.026) and recessive model del/del vs. ins/del + ins/ins (*P_Q_* = 0.028) in the overall population, Asians, population-based studies, and high quality studies were not material changed by omitting this study, indicating that our results were robust and reliable. The results indicated that the study Sun et al. [8] was the major source of the heterogeneity in the meta-analysis.

Some limitations of this meta-analysis should be addressed. First, in subgroup analysis by ethnicity, the included studies regarded only Asians and Caucasians. Data concerning other ethnicities such as Africans were not found. Thus, additional studies are warranted to evaluate the effect of this functional polymorphism on CRC risk in different ethnicities, especially in Africans. Second, our results were based on unadjusted estimates. We did not perform analysis adjusted for other covariates such as smoking, drinking, obesity, red meat consumption, and so on, because of the unavailable original data of the eligible studies.

In conclusion, our meta-analysis provided a more precise estimation based on larger sample size compared with the individual studies. Our study suggested that the CASP8 is a candidate gene for CRC susceptibility. The CASP8 −652 6N ins/del polymorphism may play a protective role in CRC development especially among Asians. In order to further verify our findings, large well designed epidemiological studies are warranted.

## Supporting Information

Figure S1 **Flow diagram of included studies for this meta-analysis.**
(TIF)

Checklist S1 (DOC)
