# Supplementary figures and images for: CASP8 -652 6N Del Polymorphism Contributes to Colorectal Cancer Susceptibility: Evidence from a Meta-Analysis
Source: PLoS One. 2014 Feb 3;9(2):e87925. doi: 10.1371/journal.pone.0087925 (PMC3912176; doi:10.1371/journal.pone.0087925)

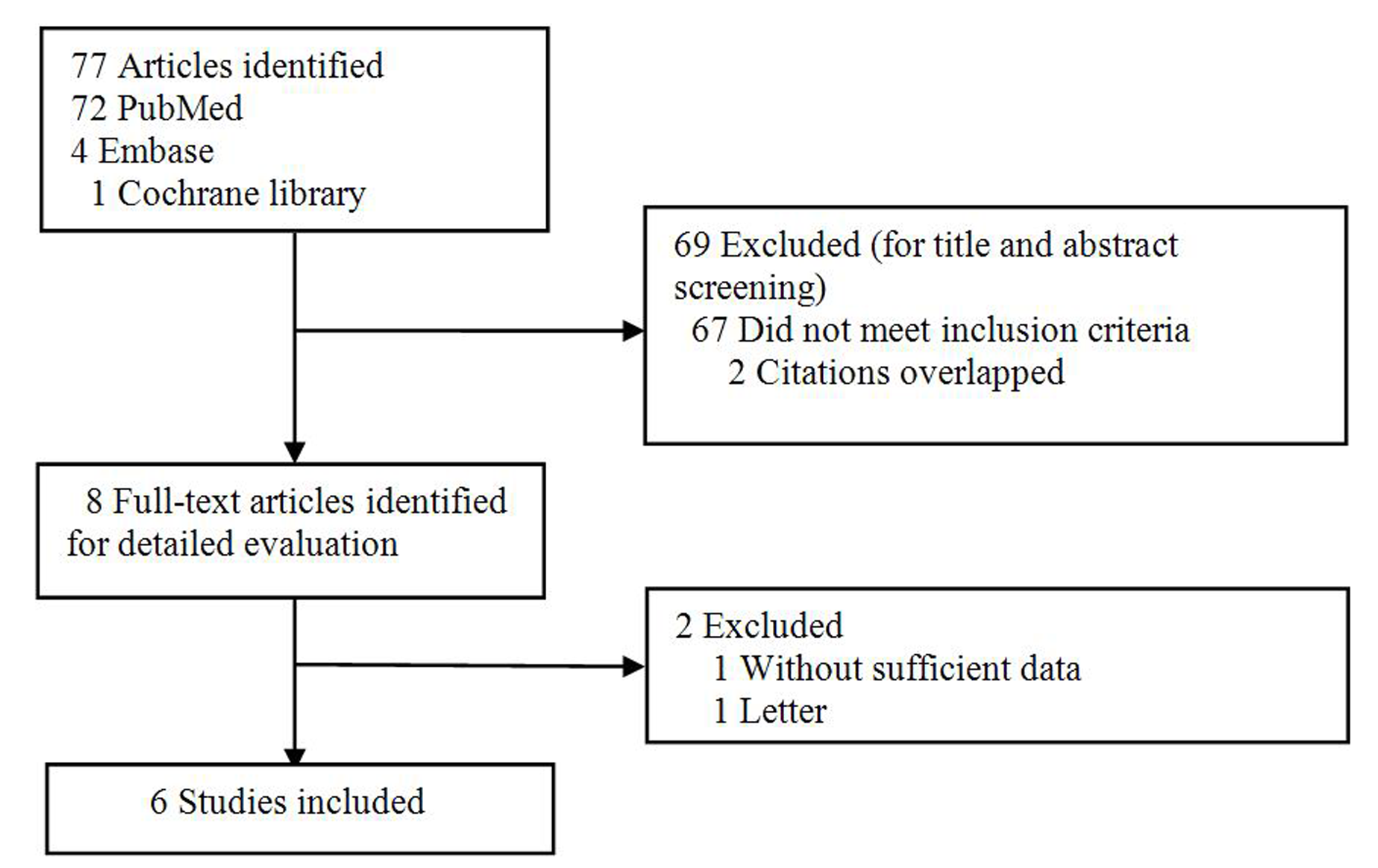

Supplement: Figure S1 — Flow diagram of included studies for this meta-analysis. (TIF) [file pone.0087925.s001.tif]
